# Single cells and transposable element heterogeneity in stem cells and development

**DOI:** 10.1186/s13619-021-00085-5

**Published:** 2021-07-06

**Authors:** Andrew P. Hutchins

**Affiliations:** 1 Shenzhen Key Laboratory of Gene Regulation and Systems Biology, Southern University of Science and Technology, Shenzhen, 518055 China; 2 Department of Biology, School of Life Sciences, Southern University of Science and Technology, Shenzhen, 518055 China

## Abstract

Recent innovations in single cell sequencing-based technologies are shining a light on the heterogeneity of cellular populations in unprecedented detail. However, several cellular aspects are currently underutilized in single cell studies. One aspect is the expression and activity of transposable elements (TEs). TEs are selfish sequences of DNA that can replicate, and have been wildly successful in colonizing genomes. However, most TEs are mutated, fragmentary and incapable of transposition, yet they are actively bound by multiple transcription factors, host complex patterns of chromatin modifications, and are expressed in mRNAs as part of the transcriptome in both normal and diseased states. The contribution of TEs to development and cellular function remains unclear, and the routine inclusion of TEs in single cell sequencing analyses will potentially lead to insight into stem cells, development and human disease.

## Main text

**Fig. 1:**
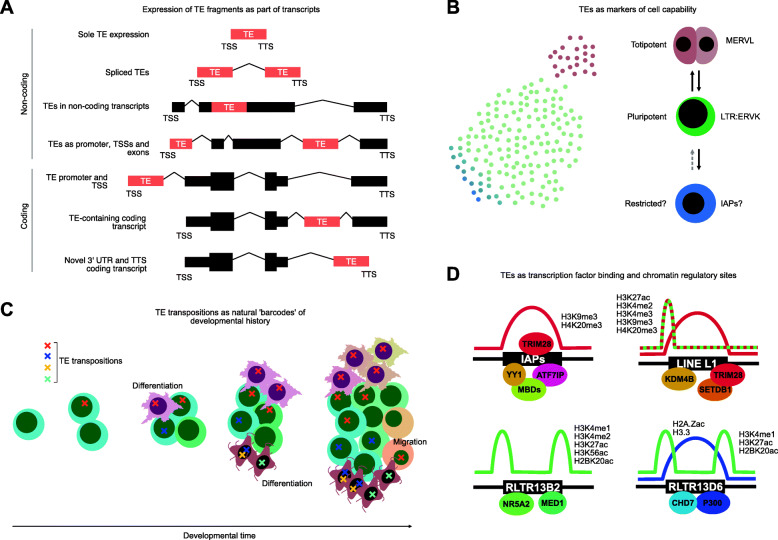
TEs and single cells, strategies to understand and exploit their properties. **a** Inactive TEs in the genome can be expressed as mRNAs. They can take several forms: as unspliced individual TE units, spliced TEs, as part of spliced or unspliced noncoding transcripts, or as part of coding transcripts, as unique promoters or inside UTRs (untranslated regions). **b** TEs are expressed in a cell type-specific manner, and can be markers for cell type specification. Shown is a schematic of the situation in a mouse PSC culture. Most cells are pluripotent, and express various ERVKs, a small number of cells express MERVL and have some totipotent properties, whilst some cells express IAPs, which may be associated with reduced developmental potency. **c** TE transposition to infer developmental lineages. Transposition of TEs can act as natural ‘barcodes’ to track the developmental lineage of adult human cells. As somatic variation is introduced during development it is propagated during subsequent cell divisions. Ultimately the pattern of TE insertions could be sequenced in single cells, and coupled with spatial transcriptomics could generate a detailed developmental roadmap for human tissues, where genetic engineering of artificial barcode systems is unavailable. **d** TEs harbor transcription factor binding sites, and recruit specific transcription factors and chromatin modifiers to modify the epigenetic state at TEs. These examples are taken from binding patterns observed in mouse PSCs

TEs are self-replicating sequences of DNA that have colonized nearly 50% of typical mammalian genomes, and take up more DNA sequence than the exons of coding genes. TEs that successfully duplicate during embryogenesis or in the germ cells can potentially enter the next generation. Consequently, TEs are particularly active during embryogenesis. However, over evolutionary time TEs lose their ability to replicate, through a mixture of sequence mutations, defective copying of TEs, and suppression by transcriptional and epigenetic silencing. Consequently, the vast majority of TEs in the human genome are not capable of transposition. Yet, despite being molecular fossils, there is a growing body of evidence that these fossil TEs are involved in normal developmental processes, including stem cells (Wang et al. 2020). TEs maintain complex patterns of chromatin modifications, and can be transcribed into mRNAs and form parts of other coding or noncoding transcripts (Bourque et al. 2018) (Fig. 1a). These effects can have functional impacts on embryonic development. For example, knocking down mRNAs containing LINE L1 sequences leads to arrest at the embryonic 2-cell stage, as LINE L1-containing mRNAs work with Nucleolin, and Trim28/Kap1 to suppress *Dux* expression, and allow embryos to exit the 2-cell stage (Percharde et al. 2018). TE-containing RNAs are expressed in both a TE-type and a stage-specific manner during embryonic development. For example, in mouse and human embryogenesis each embryonic stage has a distinct pattern of TE expression (Goke et al. 2015; Wang et al. 2020). These patterns can be recapitulated in mouse pluripotent stem cells (PSCs). Mouse PSCs typically resemble the early epiblast in both gene and TE expression, but a small percentage of cells in mouse PSC cultures express a mouse-specific endogenous retrovirus (ERV), MERVL, which is also specifically expressed in 2-cell stage mouse embryos. Intriguingly, MERVL expressing cells have some totipotent properties, and gene expression reminiscent of 2-cell stage embryos (Macfarlan et al. 2012) (Fig. 1b), and there are indications that MERVL mRNAs are functionally important in entry and exit from the 2-cell embryonic stage. TE expression can act as a molecular characteristic to define different states of human PSCs. For example, the human-specific HERVH is expressed in primed hPSCs that resemble the early epiblast, whilst the SVA family of TEs are expressed in naïve hPSCs that resemble the inner cell mass (Theunissen et al. 2016). TEs may thus be a useful marker to explore sub-cell types with enhanced or restricted capabilities within cell cultures (Fig. 1b). TEs are also widely expressed in post implantation tissues, and two recent studies explored the expression patterns of TEs in mouse gastrulation using single cell RNA-seq data (He et al. 2021; Shao and Wang 2021). Both studies observed TE-type specific expression restricted to developmental lineages, and TE expression had especially complex patterns in extraembryonic tissues.

Whilst the vast majority of TEs are inactive, a few families of TEs are active in humans and mice. These ‘hot’ TEs retain the ability to transpose, and can introduce variance into the genome. The LINE L1 family is active in humans, and during embryogenesis several TE duplications are potentially introduced into single cells of the germ line, resulting in mosaic germ cells with novel TE insertions (Faulkner and Billon 2018). However, extraembryonic and somatic tissues are also sites of TE activity (Chuong 2018), and potentially transposition (Faulkner and Billon 2018). In humans, LINE L1s are particularly active in the extraembryonic and possibly gastrulating embryonic tissues, and LINEs are also active in the developing brain. When LINE L1s duplicate during brain development they are passed to their progeny, leading to mosaicism which could be used to trace the history of cell division and so the developmental lineage of the brain (Fig. 1c). An ingenious study took advantage of the somatic mosaicism of the LINE L1-family of TEs in the human brain, although limitations in technology at the time made it possible to look at  only a small number of neurons (Evrony et al. 2015). However, innovations in spatial single cell RNA/DNA-seq, which preserves the location of a cell in its tissue, coupled to sequencing of novel retrotransposition events, could lead to a detailed developmental roadmap of the human brain. Whilst not currently feasible in a complete adult organism at the single cell level, technological innovations will eventually make this a viable strategy.

Nonetheless, the analysis of TEs is fraught with difficulty both for measuring TE transcript expression and novel TE insertions. Indeed, estimates of transposition rates in neuronal cells range widely from 0.04 per cell to 13.7 per cell (Faulkner and Billon 2018). The large range is due to problems in accurately sequencing novel insertions in the genome. Similar issues trouble the measurement of inactive TE sequence fragments spliced into transcripts (Babarinde et al. 2019). A common solution is to combine all TE copies of the same type into a single ‘meta-element’ that represents the activity across the genome. This approach can be helpful in single cell analysis, where mapping of short reads to TEs is difficult, and data sparsity remains a challenge. Nonetheless, combining TEs into a single meta-element sacrifices important TE information at specific genomic loci, which could be exploited to understand biological phenomena, such as TE functions at specific genes or regulatory regions. However, the uncertainties in identifying the precise genomic locus of a TE sequence in the DNA has hampered the discovery of simple relationships between a TE and a gene, and may explain the relative paucity of these specific relationships in the literature. An interesting study utilized transcript assembly to improve this aspect in single cell expression analysis (Shao and Wang 2021). In that study TEs were first assembled into transcripts using bulk short-read RNA-seq, and then the single cell RNA-seq data was mapped to the assembled transcripts. Their work can potentially place expressed TEs in their genomic context, which can unlock important information. However, assembling transcripts together from short reads is difficult to do accurately, even in species with robust genome and transcriptome annotations (Babarinde et al. 2019). A possible solution involves the application of long-read and single-molecule sequencing technologies to assemble full-length TE containing transcripts in single cells. A recent study exploited both short and long-reads to identify splicing patterns in single cells of mouse embryonic brain, although TEs were not addressed (Lebrigand et al. 2020). One complication however, is that long and short reads have both advantages and disadvantages: short reads have excellent dynamic range, but are poor at assembling transcripts, whilst, conversely, long reads are excellent at assembling transcripts, but have weak dynamic range, and can detect extremely rare, possibly spurious transcripts. Ultimately, some combination of long and short reads applied to sc-RNA-seq will be a powerful technique to exploit information from TE sequences in mRNAs.

When TEs are transpositionally active, they compete with the cell transcriptional machinery, and often contain transcription factor (TF) binding sites that the TE exploits to promote their own transcription and so transposition. Hence TEs can and do act as promoters and transcription start sites. In pluripotent stem cells the long terminal repeats (LTRs) from several ERV families act as pluripotent-specific transcription start sites (Fort et al. 2014). TEs thus exploit the endogenous transcriptional machinery to promote their own expression by containing TF binding sites for pluripotency TFs (Wang et al. 2020). During evolution, as TEs transpose, they shuffle the enhancer elements controlling pluripotency genes. This helps explain the dramatic differences in genome binding sites of two pluripotency TFs, OCT4 and NANOG, in mouse and human PSCs. Despite ostensibly performing the same function and regulating similar sets of genes, OCT4 and NANOG binding sites are substantially different between mouse and human PSCs, most likely due to the activity of TEs (Kunarso et al. 2010). This property of TEs can also help explain why TEs are expressed in somatic cells. Each TF in a family can bind a similar DNA sequence, yet different members of the same TF family can be active in widely divergent cell types and tissues, hence a TF that regulates a TE during embryogenesis may have a corresponding family member in somatic tissues that can activate the same TE. This may help explain the surprising widespread expression and activity of TEs even in somatic tissues, particularly the brain and the immune system (Faulkner and Billon 2018; He et al. 2021). A consequence of TFs binding to TEs is the presence of TE-type specific chromatin modifications (He et al. 2019) (Fig. 1d). Some TE types are silenced by histone methylations, whilst others have active histone acetylation, and yet further TEs are bivalently marked with by both methylation and acetylation. Almost certainly divergent and combinatorial patterns of TFs binding to TEs is driving this effect. However, much remains to be discovered, as all of the above analysis was performed in pooled cells, meaning that TF and chromatin behavior in single cells at TEs remains unclear. Innovations in single cell methods that probe the epigenetic state (e.g. single cell chromatin accessibility or DNA methylation) may lead to insight into TF binding to TE sequences, and the consequences for regulation of the epigenome.

Ultimately, TEs are an integral part of the transcriptional output of cells, are major sites of chromatin regulation, and contribute to cell type heterogeneity in unclear ways. An Improved understanding of TE activity in single cells, as transcribed units, chromatin elements, and transposition events, will lead to insight into cellular function.
